# Characterization of chromosome composition of sugarcane in nobilization by using genomic in situ hybridization

**DOI:** 10.1186/s13039-018-0387-z

**Published:** 2018-06-07

**Authors:** Fan Yu, Ping Wang, Xueting Li, Yongji Huang, Qinnan Wang, Ling Luo, Yanfen Jing, Xinlong Liu, Zuhu Deng, Jiayun Wu, Yongqing Yang, Rukai Chen, Muqing Zhang, Liangnian Xu

**Affiliations:** 1 National Engineering Research Center for Sugarcane, Fujian Agriculture and Forestry University, Fuzhou, China; 2 Guangdong Provincial Bioengineering Institute, Guangzhou Sugarcane Industry Research Institute, Guangzhou, China; 3 Sugarcane Research Institute of Yunnan Agriculture Science Academy, Kaiyuan, China; 4 Guangxi Collaborative Innovation Center of Sugar Industries, Guangxi University, Nanning, China

**Keywords:** *Saccharum officinarum*, *Saccharum spontaneum*, Interspecific hybridization, Genomic in situ hybridization (GISH), Chromosome transmission

## Abstract

**Background:**

Interspecific hybridization is an effective strategy for germplasm innovation in sugarcane. Nobilization refers to the breeding theory of development and utilization of wild germplasm. *Saccharum spontaneum* is the main donor of resistance and adaptive genes in the nobilization breeding process. Chromosome transfer in sugarcane is complicated; thus, research of different inheritance patterns can provide guidance for optimal sugarcane breeding.

**Results:**

Through chromosome counting and genomic in situ hybridization, we found that six clones with 80 chromosomes were typical *S. officinarum* and four other clones with more than 80 chromosomes were interspecific hybrids between *S. officinarum* and *S. spontaneum*. These data support the classical view that *S. officinarum* is characterized by 2n = 80. In addition, genomic in situ hybridization showed that five F_1_ clones were products of a 2n + n transmission and one F_1_ clone was the product of an n + n transmission in clear pedigree noble hybrids between *S. officinarum* and *S. spontaneum*. Interestingly, Yacheng 75–408 and Yacheng 75–409 were the sibling lines of the F_1_ progeny from the same parents but with different genetic transmissions.

**Conclusions:**

This is the first clear evidence of Loethers, Crystallina, Luohanzhe, Vietnam Niuzhe, and Nanjian Guozhe were typical *S. officinarum* by GISH. Furthermore, for the first time, we identified the chromosome transmission of six F_1_ hybrids between *S. officinarum* and *S. spontaneum*. These findings may provide a theoretical basis for germplasm innovation in sugarcane breeding and guidance for further sugarcane nobilization.

**Electronic supplementary material:**

The online version of this article (10.1186/s13039-018-0387-z) contains supplementary material, which is available to authorized users.

## Background

Sugarcane, which belongs to the genus *Saccharum* in the family *Poaceae* and the tribe *Andropogoneae*, is related to *Miscanthus*, *Sclerostachya*, *Erianthus*, and *Narenga*, and constitutes the *Saccharum* complex. The genus *Saccharum* comprises six species, including *Saccharum officinarum*, *Saccharum robustum*, *Saccharum spontaneum*, *Saccharum sinense*, *Saccharum barberi*, and *Saccharum edule* [1]. Of these, *S. spontaneum* and *S. robustum* are considered to be wild species, as the others have been cultivated [2]. Except for *S. edule*, five other native species, including *S. officinarum* (2n = 80) and *S. spontaneum* (2n = 40–128), have played an important role in sugarcane breeding [1]. *S. officinarum* (which is referred as “noble” cane) is essential for sugarcane breeding program, as it is the main source of alleles controlling high sugar content and almost all modern sugarcane cultivars contain its lineage [3]. Typically, *S. officinarum* have 2n = 80 chromosomes [4], with a basic chromosome number of x = 10 [5]. *S. spontaneum* is a wild species characterized by high stress-resistance, and then is the most valuable wild germplasm resources in the genus *Saccharum* [6]. It has a wide range of chromosome numbers, ranging from 2n = 40 to 128 [7, 8]. Recently, research on *S. sinense* and *S. barberi* has shown that they are derived from natural interspecific hybridization between *S. officinarum* and *S. spontaneum* [9]. Furthermore, all modern sugarcane cultivars were hybrids between *S. officinarum* and *S. spontaneum* in the twentieth century [10]. The first artificial interspecific hybrids between these two species were created to overcome disease outbreaks and were followed by repeated backcrossing using *S. officinarum* as the recurrent female parent to restore high sucrose content. This procedure is referred as “nobilization”.

Interspecific hybridization is an innovative and effective method for sugarcane breeding. This strategy allows for increasing stress-resistance from *S. spontaneum*, as well as maintaining high sugar genes from *S. officinarum*, which promote the genetic improvement process [3]. Through the process of sugarcane nobilization, utilization of diverse clones of *S. officinarum* and *S. spontaneum* has been proposed as a way to introduce genetic diversity [11, 12]. While a large number of germplasm resources are available for exploitation, a limited understanding of the quantitative aspects of nobilization makes the parent selection process for nobilization difficult. In 1922, Bremer discovered the classical cytological peculiarity of 2n chromosome transmission from *S. officinarum* in interspecific crosses with *S. spontaneum* [5]. Later studies verified his work and further demonstrated that the same process occurs in BC_1_ when *S. officinarum* is used as the female parent [13]. Endoduplication, or fusion of two nuclei following the second meiosis, has been proposed by Bhat and Gill to explain this peculiar chromosome transmission [14]. However, Roach found that n + n transmission occurs in crosses between *S. officinarum* and *S. spontaneum* with 2n = 80, but seldom occurs in crosses between *S. officinarum* and *S. spontaneum* with 2n = 64 or 96 [15].

Modern sugarcane cultivars are derived from intercrossing between the first nobilized hybrids of a few parental clones with chromosome numbers ranging from 100 to 130, approximately 10% of which originating from *S. spontaneum* [5, 15]. The accurate number of *S. spontaneum* chromosomes in the different cultivars is not completely understood, as is their segregation during successive crosses. This problem impedes our understanding of the exact genetic contribution of *S. spontaneum* to sugarcane cultivars. To innovate germplasm in sugarcane breeding, study on chromosome composition of the progenies between *S. officinarum* and *S. spontaneum* in sufficient early generation is needed. Genomic in situ hybridization (GISH) is a highly efficient molecular cytogenetic tool that takes genomic DNA from one species as the labelled probe in hybridization experiments to chromosomal DNA in situ [16, 17]. The technique is mainly used to identify chromosome recombination, genetic relationship of interspecific hybrids, and chromosome transmission [5, 18]. To date, many researches had verified the accuracy and high-efficiency of the GISH technology in studying the chromosome composition and chromosomal translocation in a wide range of natural allopolyploids or artificial polyploidy progenies [19–22]. D’Hont et al., for the first time, demonstrated that GISH can be used to differentiate parental chromosomes in interspecific hybrids between BNS 3066 (*S. officinarum*) and SES 14 (*S. spontaneum*) [5, 18]; in addition, they identified n + n transmission of parental chromosomes in the interspecific F_1_ between *S. officinarum* and *S. spontaneum*. They also analyzed chromosomes of cultivar “R570” and found that approximately 10% originated from *S. spontaneum* and another approximately 10% were recombinant chromosomes, demonstrating that exchanges had occurred between chromosomes derived from *S. officinarum* and *S. spontaneum*. Recently, George Piperidis et al. used GISH to identify the occurrence of 2n + n transmission in crosses and the first backcrosses of *S. officinarum* and *S. spontaneum* [4]. GISH was also applied to identify parental genomes of an intergeneric hybrid between *S. officinarum* and a related wild species, *Erianthus arundinaceus*. These studies confirmed that the F_1_ and BC_2_ crosses resulted from an n + n chromosome transmission, while the BC_1_ cross resulted from a 2n + n transmission [23].

To date, most modern sugarcane cultivars are derived from a few clones of *S. officinarum*. The limited number of parents have leaded to narrow genetic background of sugarcane, various *S. officinarum* should be identified for germplasm innovation. Additionally, clear chromosome composition of early progeny between *S. officinarum* and *S. spontaneum* will provide enough valid germplasm for further sugarcane nobilization. The aim of the present study was to verify the authenticity of ten clones classified as *S. officinarum* via chromosome counting and GISH. Six clear pedigree noble F_1_ chromosome constitutions were analyzed using GISH. Our results will be applied to select the purest *S. officinarum* and valid germplasm for sugarcane breeding.

## Results

### Chromosome counting for identification of the authenticity of *S. officinarum*

**Fig. 1:**
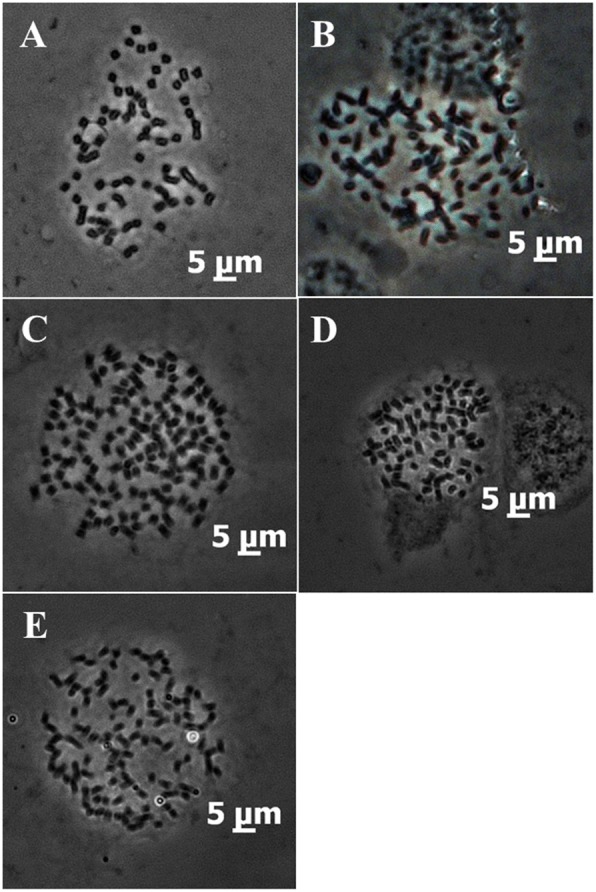
The metaphase chromosomes of five clones of sugarcane. **a**: Badila; **b**: 50uahapele; **c**: Muckche; **d**: Luohanzhe; **e**: Baimeizhe

We obtained chromosome preparations suitable for counting chromosomes in ten clones classified as *S. officinarum* (Table 2). The chromosomes were well spread with little cytoplasm background in all materials. Partial results are shown in Fig. 1, the rest results are shown in Additional file 1: Figure S1. The modal number of chromosomes for Muckche, Canablanca, 50uahapele, and Baimeizhe was 2n > 80, ranging from 86 to 114 (Table 2); however, in others six clones the chromosome modal number was 2n = 80.

### GISH for identification of the authenticity of *S. officinarum*

**Fig. 2:**
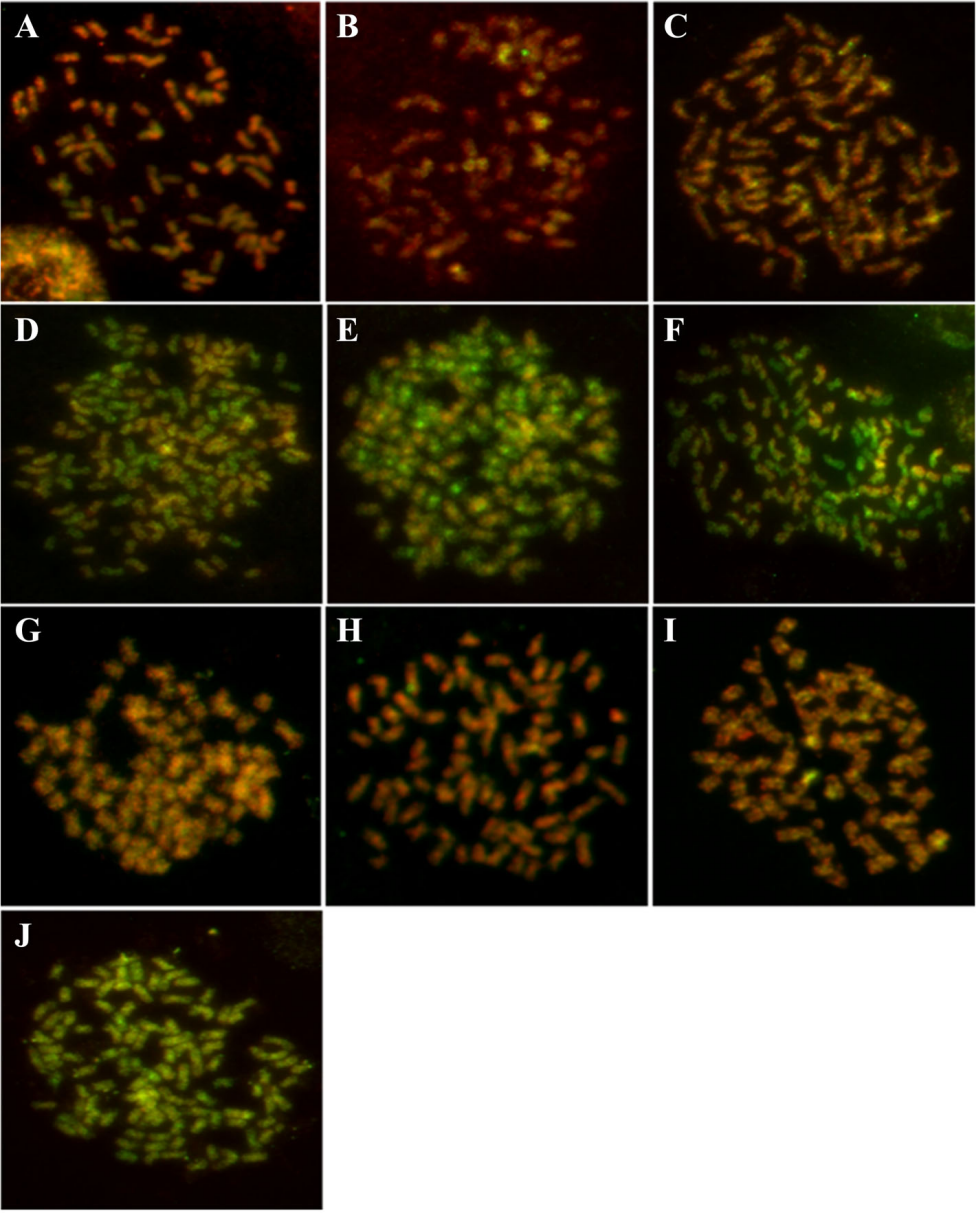
GISH results of ten *S. officinarum* clones using biotin labelled *S. officinarum* genomic DNA and digoxigenin labelled *S. spontaneum* genomic DNA. **a**: Badila; **b**: Loethers; **c**: Crystallina; **d**: Muckche; **e**: Canablanca; **f**: 50uahapele; **g**: Luohanzhe; **h**: Vietnam Niuzhe; **i**: Nanjian Guozhe; **j**: Baimeizhe; The chromosomes of *S. officinarum* show orange-yellow fluorescent, while those of *S. spontaneum* show green-yellow fluorescent

GISH was carried out on the metaphase chromosomes of ten clones classed as *S. officinarum*. In chromosomes, sequences homologous to *S. officinarum* total DNA fluoresced red and sequences homologous to *S. spontaneum* total DNA fluoresced green. However, due to the high homology of *S. officinarum* and *S. spontaneum* genomes, *S. officinarum*-derived and *S. spontaneum*-derived chromosomes were visualized in orange-yellow and green-yellow, respectively. The chromosomes of ten clones classed as *S. officinarum* were labeled in orange-yellow and green-yellow, respectively. The fluorescence of the two groups of chromosomes were differentially enhanced where their sequences were different, orange or green (Fig. 2d, e, f, and j).

In Badila, Loethers, Crystallina, Luohanzhe, Vietnam Niuzhe, and Nanjian Guozhe clones, all chromosomes fluoresced orange-yellow, indicating that the red signals were stronger than the green signals (Fig 2a, b, c, g, h, and i). These materials derived from only *S. officinarum* lineage. However, according to the color, the chromosomes of Muckche, Canablanca, 50uahapele, and Baimeizhe can be identified as two groups, orange-yellow and green-yellow (Fig 2d, e, f, and j). These orange-yellow chromosomes were derived from *S. officinarum*. While, the rest chromosomes fluoresced green-yellow were derived from *S. spontaneum*. Thus, these materials were hybrids between *S. officinarum* and *S. spontaneum*.

### GISH of F_1_ hybrids between *S. officinarum* and *S. spontaneum*

**Fig. 3:**
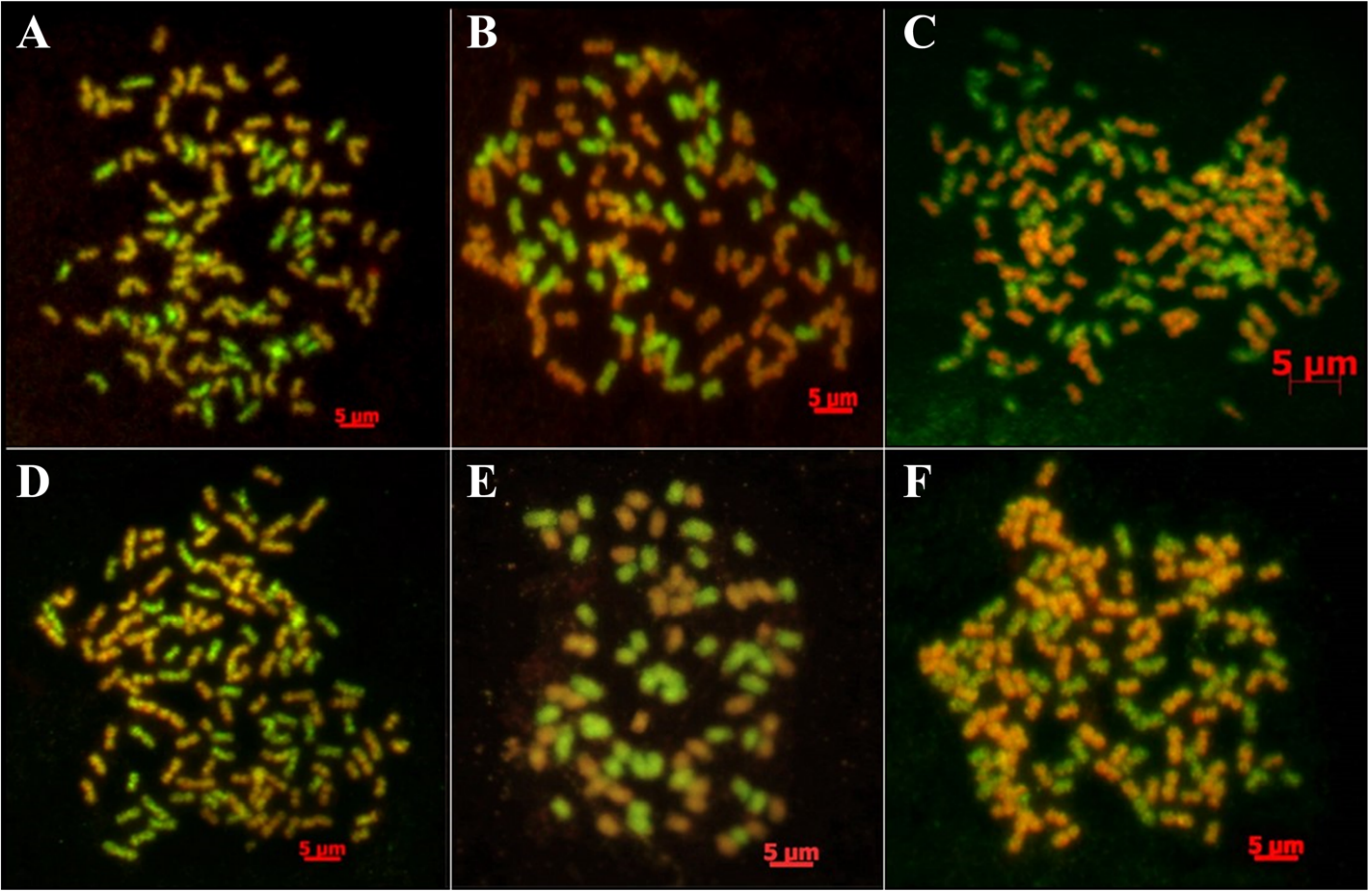
GISH results of six F_1_ hybrids between *S. officinarum* and *S. spontaneum* using biotin labelled *S. officinarum* genomic DNA and digoxigenin labelled *S. spontaneum* genomic DNA. **a**: Yacheng 82–108; **b**: Yacheng 58–43; **c**: Yacheng 58–47; **d**: Yacheng 75–419; **e**: Yacheng 75–408; **f**: Yacheng 75–409

In the six F_1_ hybrids analyzed, five F_1_ hybrids, including Yacheng 82–108, Yacheng 58–43, Yacheng 58–47, Yacheng 75–409, and Yacheng 75–419, had 2n = 112 or 120, of which 80 were derived from the *S. officinarum* female parent, and *n* = 32 or *n* = 40 derived from the male parents of *S. spontaneum*, being consistent with a typical 2n + n transmission of parental chromosomes (Table 3; Fig. 3a, b, c, d, f). However, Yacheng75–408, a sibling line of Yacheng 75–409 from the same parental combination, had 2n = 80, of which 40 were derived from the *S. officinarum* female parent and the other 40 were derived from the *S. spontaneum* male parent (Table 3; Fig. 3e). Therefore, Yacheng 75–408 is consistent with an n + n transmission of parental chromosomes.

## Discussion

### The authenticity of *S. officinarum*

Modern sugarcane cultivars have complex and unique genome structures and variable chromosome numbers. *S. officinarum*, which includes Badila, Black Cheribon, Crystallina, and Otaheite, has 2n = 80 chromosomes [24]; those with more than 80 chromosomes are likely to be hybrids [13, 24]. Badila is commonly used for sugarcane breeding and sugar production. Previous studies have indicated that of 31 clones in New Guinea, 29 were typical clones with chromosome number of 2n = 80 and two were atypical clones with chromosome number of 2n = 116 and 70 [25]. Piperidis et al. showed that six atypical clones (2n > 80) belong to hybrids from *S. officinarum* and *S. spontaneum*, indicating that more than 80 chromosome clones may not have originated from a pure *S. officinarum* [4]. In our study, the chromosome numbers of 50uahapele (2n ≈ 86), Muckche (2n ≈ 142), Baimeizhe (2n ≈ 104) and Canablanca (2n ≈ 114) were exceeded 80. Then, using GISH, we demonstrated that these cultivars were hybrids with a portion of chromosomes derived from *S. spontaneum*. Hence, these results were consistent with previous reports that *S. officinarum* may be characterized by 2n = 80 [26]. Furthermore, these differential typical *S. officinarum* will broaden the narrow genetic of sugarcane and provide larger pure *S. officinarum* for selecting cross parents in nobilization.

### Chromosome transmission in F_1_ hybrids between *S. officinarum* and *S. spontaneum*

Interspecific hybridization had proved to be a major breakthrough for germplasm innovation in sugarcane breeding. POJ2878 is one of the most successful example in nobilization that has been widely applied [27]. However, the practical chromosome transmission is crucial for obtaining an ideal species with higher sugar, higher yield, and greater stress-resistance in nobilization. Diversity of chromosome transmission in F_1_ hybrids had deeply affected the efficiency of sugarcane breeding. Different genetic inheritance would lead to diverse traits of the progeny. Cytogenetic studies have demonstrated that 2n + n chromosome transmission can occur in crosses between *S. officinarum* (female) and *S. spontaneum* (male); this was also confirmed by Piperidis [4]. The 2n + n transmission is key to the nobilization process since it accelerates return to the sugar-producing type. However, the results of chromosome counting showed that n + n transmission often occurs with crosses of *S. spontaneum* with 2n = 80 as a male parent and seldom in crosses of *S. spontaneum* with 2n = 64 and 96 [15]. D’Hont et al. revealed n + n transmission of parental chromosomes by using GISH to analyze an interspecific hybrid between *S. officinarum* (2n = 80) and *S. spontaneum* (2n = 64) [5]. Here, we confirmed that four F_1_ clones of different series (*S. spontaneum* with 2n = 80 or 2n = 64 as male parents) were 2n + n. Furthermore, two different nobilization F_1_ clones of the same series had two different transmissions simultaneously, 2n + n or n + n, with *S. spontaneum* (2n = 80) as the parent. Altogether, these results concluded that diverse transmissions, 2n + n or n + n, will occurs in two different ploidy *S. spontaneum* (2n = 80 or 2n = 64 as male parents). Therefore, different ploidy *S. spontaneum* have no influence on the type of chromosome transmission (2n + n versus n + n). Furthermore, nobilization may produce different frequencies of n + n, 2n + n, and aneuploid offspring in larger numbers of F_1_ clones.

Many studies have shown that most F_1_ crosses and BC_1_ backcrosses result in chromosome doubling of the noble parent *S. officinarum* in transmission with the 2n chromosome [6–8, 28]. Although 2n + n is the main chromosome transmission in nobilization, there are also cases of n + n transmission [4, 5, 29]. Even more, in our study, we found that the differential transmissions in the same parents using GISH. Indeed, chromosome transmission is complex in sugarcane and further studies should be performed to guide optimized sugarcane breeding. The 2n + n chromosome transmission in interspecific crosses is considered an important factor in the rapid breakthrough that interspecific hybridization has provided to sugarcane breeding, leading to a rapid reduction in the proportion of chromosomes from wild species of hybrids and subsequent backcrosses to rapidly recover clones with highest sugar content [3, 30].

## Conclusions

Using GISH, this is the first direct evidence that Loethers, Crystallina, Luohanzhe, Vietnam Niuzhe, and Nanjian Guozhe with 80 chromosomes were typical *S. officinarum*; while 50uahapele, Muckche, Baimeizhe and Canablanca with more than 80 chromosomes were interspecific hybrids between *S. officinarum* and *S. spontaneum*. Additionally, GISH analysis demonstrated that five F_1_ hybrids between *S. officinarum* and *S. spontaneum*, Yacheng 82–108, Yacheng 58–43, Yacheng 58–47, Yacheng 75–409, and Yacheng 75–419 were products of a 2n + n transmission; while, Yacheng 75–408 was the product of an n + n transmission. Although Yacheng 75–408 and Yacheng 75–409 are the sibling lines of the different F_1_ progeny with the same parents, there was a large difference in chromosome numbers that led to different patterns of chromosome inheritance. The results of this study support previous reports that *S. officinarum* may be characterized by 2n = 80 and provide more useful molecular cytogenetic information for the larger germplasm resources of *S. officinarum*. Futhermore, clear chromosome composition of early progenies between *S. officinarum* and *S. spontaneum* will provide guidance for further sugarcane nobilization.

## Methods

### Plant materials and DNA extraction

In this study, ten experimental materials classified as *S. officinarum* were used, including Badila, Loethers, Crystallina, 50uahapele, Muckche, Canablanca, Luohanzhe, Vietnam Niuzhe, Nanjian Guozhe, and Baimeizhe. Of these, 50uahapele, Canablanca, Baimeizhe were provided by the research Institute Ruili Station of Yunnan Agriculture Science Academy; the Sugarcane Research Institute of Yunnan Agriculture Science Academy provided the rest materials. The Hainan Sugarcane Breeding Station, Guangzhou Sugarcane Industry Research Institute provided six F_1_ clones between *S. officinarum* and *S. spontaneum* for nobilization (Table 1). All plant materials used in this study were grown in the germplasm resources nursery at the Fujian Agriculture and Forestry University. Leaf tissues from the above materials were ground in liquid nitrogen and stored at − 80 °C. Total genomic DNA was extracted from young leaves following CTAB methodology [31].

**Table 1:** Crosses of A, B, C, and D

Cross	Clone	Female (♀)	Male (♂)
A	Yacheng 82–108	Badila (2n = 80; S.o)	Yunnan 75–2-11 (2n = 64; S.s)
B	Yacheng 58–43; Yacheng 58–47	Badila (2n = 80; S.o)	Yacheng (2n = 80; S.s)
C	Yacheng 75–419	Fiji (2n = 80; S.o)	Yacheng (2n = 80; S.s)
D	Yacheng 75–408; Yacheng 75–409	Vietnam Niuzhe (2n = 80; S.o)	Yacheng (2n = 80; S.s)

*S.o S. officinarum*, *S.s S. spontaneum*

### Chromosome preparation

Root tips were obtained from ten clones classified as *S. officinarum* and six clones of F_1_ between *S. officinarum* and *S. spontaneum*. Meristem of root-tips were treated with saturated p-dichlorobenzene solution for 1.5 h at 25 °C. The root tips were then fixed in 3:1 (*v*/v) ethanol: acetic acid solution for 24 h and successive eluted in ethanol solution (75, 95 and 100% ethanol), finally kept at − 20 °C with 75% ethanol solution. The fixed roots were washed in water and digested in an enzyme solution (4% Onozuka R10 cellulose, 0.5% pectolyase Y-23 and 0.5% pectinase) for 4 h at 37 °C. The digestive meristematic cells were squashed on the clear slide in 20 μL of 3:1 (v/v) ethanol: acetic acid. Slides were stored at − 20 °C.

### Probe labelling

Probes were labelled using a Nick-translation kit with biotin-dUTP (Roche, Germany) and digoxigenin (Roche, Germany). For in situ hybridization, 100 ng/μL of Badila (*S. officinarum*) genomic DNA, labeled with biotin-dUTP, and 100 ng/μL Yunnan 75–2-11 (*S. spontaneum*) genomic DNA, labeled with digoxigenin were used as probes.

### Genomic in situ hybridization (GISH)

GISH technique were performed as described previously by D’Hont et al. [32] with moderate improvement. The denaturing solution included 70% formamide in 2× SSC. Slides were denatured in this solution for 3 min at 80 °C. Dehydration was performed in cold ethanol and slides were then air dried at room temperature. The probe mixture including hybridization buffer (50% formamide, 2× SSC, 10% dextransulfate) and 200 ng labeled probe after denaturation for 10 min at 97 °C was applied to each slide and incubated for 20 h at 37 °C in a humid dark box. The high stringency conditions of post-hybridization washes were carried out with 2 × SSC for 8 min at 42 °C, a second wash in 50% formamide, 2 × SSC, pH 7.0, for 3 × 8 min at 42 °C, followed by a rinse in 2 × SSC for 8 min at room temperature and a final wash in 0.1 × SSC for 3 × 8 min at 55 °C. The biotin-labelled probe was detected with avidin-conjugated Texas red and the digoxigenin-labelled probe was detected with FITC (fluorescein isothiocyanate)-conjugated anti-digoxigenin antibody. Slides then were counterstained with 4′, 6-diamidino-2-phenylindole (DAPI) in a Vectashield anti-fade solution (Vector Laboratories, Burlingame, CA). GISH signals were captured using the AxioVision measurement module of AxioScope A1 Imager fluorescent microscope (Zeiss, Germany).

### Chromosome counting

The metaphase chromosomes of the above materials were captured using phase contrast microscope (Fig. 1) or fluorescence microscope (Additional file 1: Figure S1; Figs. 2 and 3). The number of chromosome was counted using the program Image-pro plus 6.0 (Media Cybernetics). Results were presented as the modal number (occurred the most times among different cells in each clone) and the number of cells observed at least 30 cells for each clone (Tables 2 and 3). Additionally, at least three materials in one generation had been studied in six F_1_ clones.

**Table 2:** The chromosome numbers and ranges of ten clones in sugarcane

Clone	Total number of cells observed	Modal number of chromosomes	Range of total numbers of chromosomes
Badila	30	2n = 80	80
Loethers	30	2n = 80	80
Crystallina	30	2n = 80	80
Muckche	30	2n = 142	141–143
Canablanca	30	2n = 114	113–115
50uahapele	30	2n = 86	85–88
Luohanzhe	30	2n = 80	80
Vietnam Niuzhe	30	2n = 80	80
Nanjian Guozhe	30	2n = 80	80
Baimeizhe	30	2n = 104	104–106

Note: Since small variations in chromosome counts can occur due to the loss or the overlapping of a few chromosomes from the preparation, the modal number of chromosomes and range of total numbers of chromosomes in 2n cells are presented for the sugarcane clones analyzed

**Table 3:** Chromosome composition of six F_1_ hybrids between *S. officinarum* and *S. spontaneum* in nobilization

Cross	Clone	No. of chromosomes	No. of S.o chromosomes	No. of S.s chromosomes	Chromosome composition	Chromosome transmission	No. of cells observed
A	Yacheng 82–108	112	80	32	80 S.o + 32 S.s	2n + n	30
B	Yacheng 58–43	120	80	40	80 S.o + 40 S.s	2n + n	35
	Yacheng 58–47	120	80	40	80 S.o + 40 S.s	2n + n	38
C	Yacheng 75–419	120	80	40	80 S.o + 40 S.s	2n + n	37
D	Yacheng 75–408	80	40	40	40 S.o + 40 S.s	n + n	32
	Yacheng 75–409	120	80	40	80 S.o + 40 S.s	2n + n	41

*S.o S. officinarum*, *S.s S. spontaneum*

## Additional file

Additional file 1: **Figure S1.** The metaphase chromosomes of five clones of sugarcane. A: Lothers; B: Crystallina; C: Canablanca; D: Vietnam Niuzhe; E: Nanjian Guozhe. (TIF 9259 kb)

## Abbreviations

DAPI: 4′, 6-diamidino-2-phenylindole
FISH: Fluorescence in situ hybridization
FITC: Fluorescein isothiocyanate
GISH: Genomic in situ hybridization
