# Supplementary figures and images for: Characterization of chromosome composition of sugarcane in nobilization by using genomic in situ hybridization
Source: Mol Cytogenet. 2018 Jun 7;11:35. doi: 10.1186/s13039-018-0387-z (PMC5992832; doi:10.1186/s13039-018-0387-z)

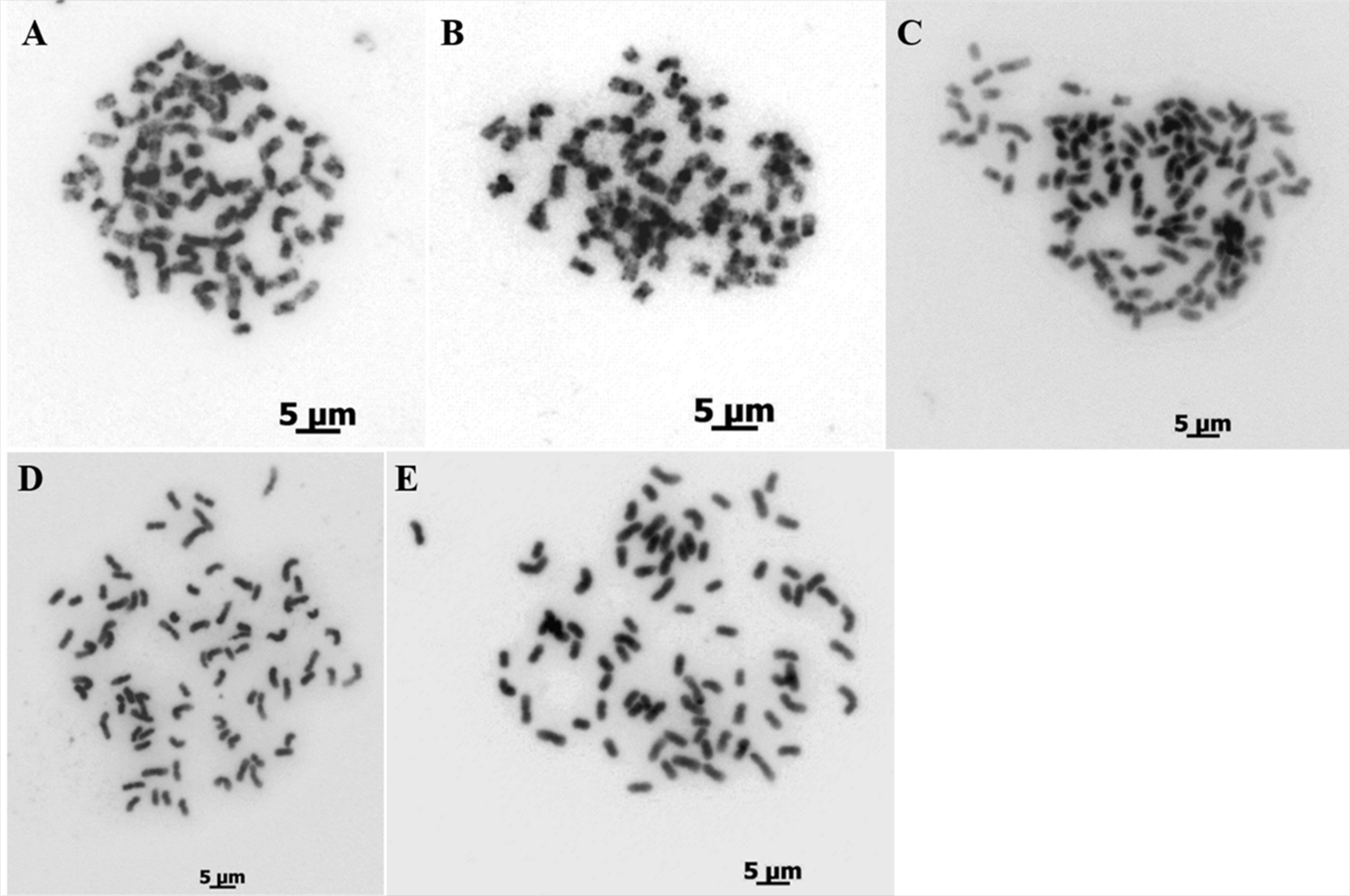

Supplement: Supplementary file 1 — Figure S1. The metaphase chromosomes of five clones of sugarcane. A: Lothers; B: Crystallina; C: Canablanca; D: Vietnam Niuzhe; E: Nanjian Guozhe. (TIF 9259 kb) [file 13039_2018_387_MOESM1_ESM.tif]
